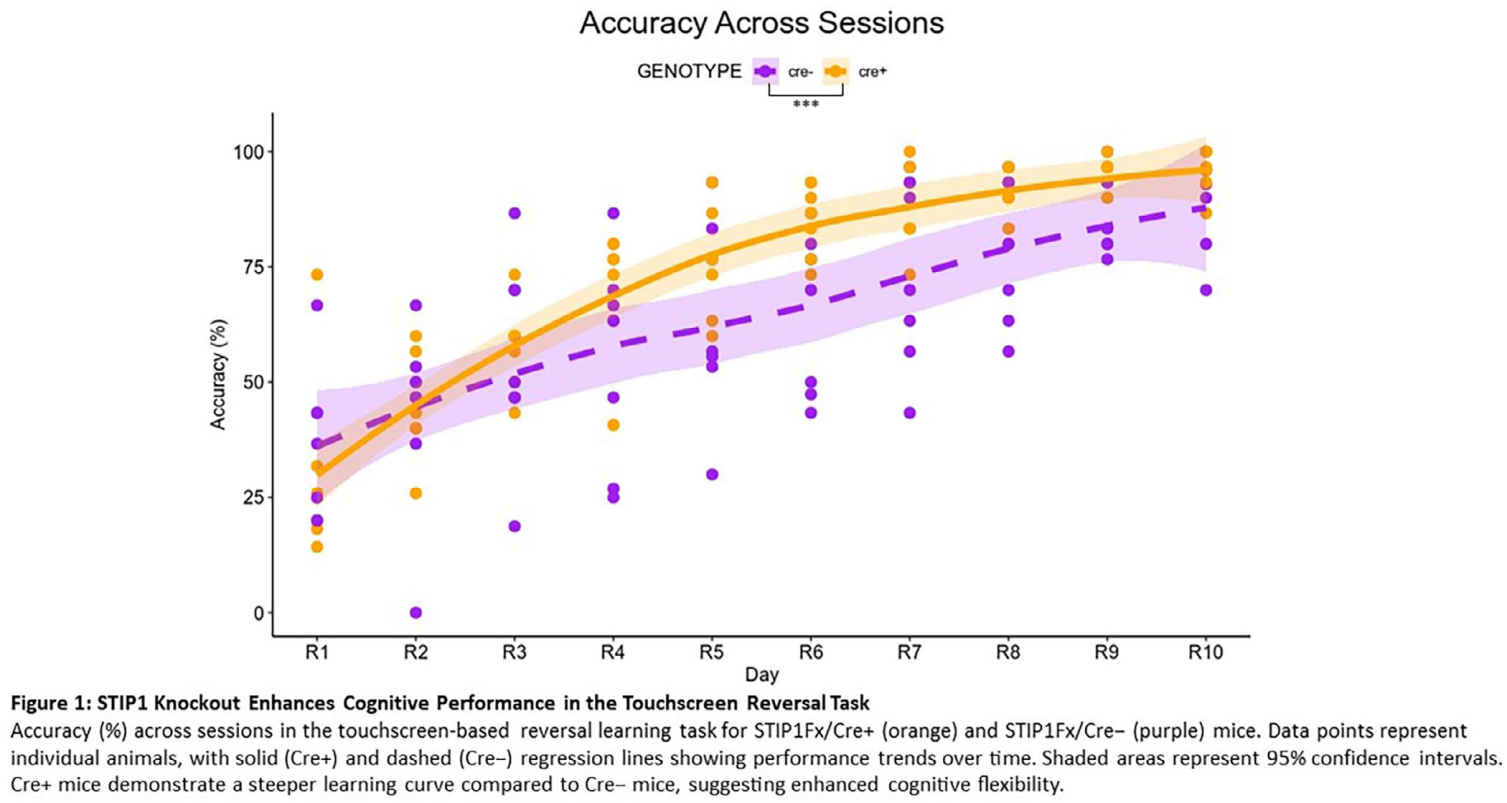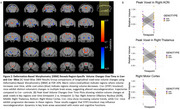# STIP1 Knockout Mitigates Disease Progression in a Synucleinopathy Mouse Model: A Multimodal Behavioral and Neuroimaging Study

**DOI:** 10.1002/alz70856_106526

**Published:** 2026-01-09

**Authors:** Sara Touj, Vladislav Novikov, Medhinee Malvankar, Piero Rodriguez, Allison Brandt, Janice Park, Stephanie Tullo, Daniel Gallino, Gabriel A. Devenyi, Timothy J Bussey, Lisa M Saksida, Ravi S. Menon, Marco AM Prado, Mallar M. Chakravarty

**Affiliations:** ^1^ Douglas Mental Health University Institute, Montreal, QC, Canada; ^2^ McGill University, Montreal, QC, Canada; ^3^ Robarts Research Institute, London, ON, Canada; ^4^ University of Western Ontario, London, ON, Canada; ^5^ Computational Brain Anatomy Laboratory ‐ Cerebral Imaging Centre ‐ Douglas Mental Health University Institute, Verdun, QC, Canada; ^6^ Cerebral Imaging Centre ‐ Douglas Mental Health University Institute, Verdun, QC, Canada; ^7^ Western University, London, ON, Canada; ^8^ Cerebral Imaging Centre, Douglas Mental Health Institute Research Centre, Montreal, QC, Canada

## Abstract

**Background:**

Synucleinopathies, including Parkinson's disease (PD), are characterized by misfolded alpha‐synuclein (αSyn) accumulation, leading to neurodegeneration, motor dysfunction, and cognitive impairments. Normally, Hsp90 helps clear misfolded proteins, but under proteostatic stress, the chaperone network forms an epichaperome, stabilizing toxic aggregates and worsening disease progression. STIP1 (Stress‐Inducible Phosphoprotein 1), a key Hsp90 (Heat Shock Protein 90) co‐chaperone, facilitates αSyn aggregation and stabilizes the epichaperome, promoting neurotoxicity. This study investigates conditional STIP1 knockout (STIP1Fx/Cre+) effects on disease progression in an αSyn mouse model, using behavioral and neuroimaging assessments. By examining cognitive outcomes alongside neurodegeneration trajectories, this work aims to determine whether disrupting the epichaperome could serve as a viable strategy for disease modification.

**Methods:**

M83 transgenic mice expressing human A53T αSyn were crossed with tamoxifen‐inducible CreER mice to generate STIP1Fx/Cre+ and STIP1Fx/Cre− littermates. At 11 weeks, mice received unilateral αSyn preformed fibrils (PFFs) injections into the right dorsal striatum to seed pathology. One week post‐surgery, mice were administered tamoxifen for five consecutive days to induce STIP1 knockout. Mice underwent touchscreen‐based cognitive assessments. Touchscreen experiments were reproduced at both at McGill and Western University. T1‐weighted magnetic resonance images (MRI; 100 μm^3^ voxels, Bruker 7T) were acquired at ‐7 and 90 days post‐injection (dpi). Deformation‐based morphometry (DBM) was used to assess voxel‐wise volumetric changes longitudinally. Linear mixed‐effects models analyzed genotype by time interactions for behavioral and MRI data.

**Results:**

STIP1Fx/Cre+ mice showed a significant genotype × day interaction (β = 1.94, *p* =  0.001) in touchscreen reversal, improving faster than STIP1Fx/Cre‐ mice (Figure 1). This suggests enhanced cognitive flexibility, replicated across sites (data shown from McGill site only).

MRI DBM analysis revealed that STIP1Fx/Cre+ mice exhibited progressive volume increases in the thalamus, motor cortex, anterior olfactory nucleus (AON), and reticular nucleus, while STIP1Fx/Cre‐ mice showed progressive atrophy (Figure 2). This suggests STIP1 knockout may influence neurodegeneration trajectories in synucleinopathy progression.

**Conclusion:**

This study provides evidence that STIP1 depletion alters disease progression, with Cre+ mice showing better behavioral outcomes and distinct neuroimaging trajectories. Larger samples and histological validation will further clarify STIP1's role and its potential as a therapeutic target for αSyn‐driven neurodegeneration.